# The leukemia associated *ETO *nuclear repressor gene is regulated by the GATA-1 transcription factor in erythroid/megakaryocytic cells

**DOI:** 10.1186/1471-2199-11-38

**Published:** 2010-05-20

**Authors:** Ram Ajore, Rakesh Singh Dhanda, Urban Gullberg, Inge Olsson

**Affiliations:** 1 Department of Hematology, C14, BMC, S-221 84 Lund, Sweden; 2 Protista Biotechnology AB, IDEON, Ole Römers väg 12, SE 223 70 Lund, Sweden

## Abstract

**Background:**

The Eight-Twenty-One (*ETO*) nuclear co-repressor gene belongs to the *ETO *homologue family also containing Myeloid Translocation Gene on chromosome 16 (MTG16) and myeloid translocation Gene-Related protein 1 (MTGR1). By chromosomal translocations ETO and MTG16 become parts of fusion proteins characteristic of morphological variants of acute myeloid leukemia. Normal functions of ETO homologues have as yet not been examined. The goal of this work was to identify structural and functional promoter elements upstream of the coding sequence of the *ETO *gene in order to explore lineage-specific hematopoietic expression and get hints to function.

**Results:**

A putative proximal *ETO *promoter was identified within 411 bp upstream of the transcription start site. Strong ETO promoter activity was specifically observed upon transfection of a promoter reporter construct into erythroid/megakaryocytic cells, which have endogeneous *ETO *gene activity. An evolutionary conserved region of 228 bp revealed potential *cis*-elements involved in transcription of *ETO*. Disruption of the evolutionary conserved GATA -636 consensus binding site repressed transactivation and disruption of the ETS1 -705 consensus binding site enhanced activity of the *ETO *promoter. The promoter was stimulated by overexpression of GATA-1 into erythroid/megakaryocytic cells. Electrophoretic mobility shift assay with erythroid/megakaryocytic cells showed specific binding of GATA-1 to the GATA -636 site. Furthermore, results from chromatin immunoprecipitation showed GATA-1 binding *in vivo *to the conserved region of the *ETO *promoter containing the -636 site. The results suggest that the GATA -636 site may have a role in activation of the *ETO *gene activity in cells with erythroid/megakaryocytic potential. Leukemia associated *AML1*-*ETO *strongly suppressed an *ETO *promoter reporter in erythroid/megakaryocytic cells.

**Conclusions:**

We demonstrate that the GATA-1 transcription factor binds and transactivates the *ETO *proximal promoter in an erythroid/megakaryocytic-specific manner. Thus, *trans*-acting factors that are essential in erythroid/megakaryocytic differentiation govern *ETO *expression.

## Background

The human ETO co-repressor family comprises the homologous nuclear proteins ETO (Eight-Twenty-One), MTG16 (Myeloid Translocation Gene on chromosome 16) and MTGR1 (Myeloid translocation Gene-Related protein1) evolutionary related to the Drosophila protein Nervy [1]. The ETO homologues do not interact directly with DNA but are recruited by transcription factors such as PLZF, BCL6, TAL1/SCL, Gfi1and Heb [2-7] to become partners of multi-protein complexes on a gene promoter [8,9]. The ETO homologues of the complexes recruit nuclear co-repressors such as N-CoR, [9-11] SIN3 [9,10,12] and SMRT [8,13], which in turn interact with histone deacetylase (HDAC) compelling transcriptional repression.

Importantly, ETO homologue genes are commonly involved in reciprocal chromosomal translocation (t) characteristic of acute leukemia. For example, the *ETO *gene becomes fused to the *AML1 *(Runx1) transcription factor gene by t(8;21) resulting in the biosynthesis of the AML1-ETO fusion protein [14,15]. Similarly, the *MTG16 *gene becomes fused to the *AML1 *gene by t(16;21) resulting in the production of the AML1-MTG16 fusion protein [16]. The oncogenic fusion proteins interfere with hematopoietic gene regulation by transcriptional repression mediated by *ETO *and *MTG16*, respectively. Co-repressors-HDAC recruited by the ETO portion of AML1-ETO diminishes chromatin accessibility leading to transcriptional repression at AML1 targets [8-10], contributing to the cellular differentiation block of the leukemic cells.

Gene expression involves both *trans*-acting factors such as transcription factors and *cis*-acting elements such as promoter, enhancer and silencer regions whose accessibility to the *trans*-acting factors is governed by the chromatin packing. The mechanisms for transcriptional on/off switching of *ETO *homologue genes have not been examined. *ETO *and *MTG16 *show distinct cell-type-specific expression suggesting differences in gene regulation. ETO is present in many normal tissues with the highest transcript level detected in brain and heart [17,18]. *MTG16 *is expressed for example in hematopoietic tissues, placenta and pancreas [18]. Furthermore, the ETO homologues are differently expressed during hematopoietic differentiation; ETO is transiently expressed during erythropoiesis, *MTG16 *is expressed in progenitor cells and downregulated during myeloid and erythroid differentiation and MTGR1 is ubiquitously expressed, further suggesting differences in gene regulation among the *ETO *homologues [19]. Results from gene targeting reveals involvement in hematopoietic development of MTG16 [20] but not of ETO [21] or MTGR1 [22]. The leukemogenic fusion protein AML1-ETO promotes self renewal of primary erythroid cells [23] concomitant with an AML-ETO-induced block of erythroid lineage commitment. This block correlates to blockade of p300/CBP coactivation complex-mediated acetylation of the erythroid regulatory transcription factor GATA-1 [24]. As *ETO *is expressed in human erythroid cells [19] it may be affected by AML1-ETO.

The restriction of hematopoietic expression of ETO to erythroid cells [19] suggests an involvement in lineage-specific gene regulation. In order to study lineage-specificity it is essential to identify structural and functional promoter elements upstream of the coding sequences of the *ETO *gene. As erythroblasts and megakaryocytes derive from a common bipotent erythroid/megakaryocyte progenitor [25] studies were done in both cell types. Our results show a critical role for an evolutionary conserved GATA binding site in transcriptional regulation of the *ETO *gene in cells of erythroid/megakaryocytic potential.

## Results

### Homologous non-coding *ETO *sequence in human, mouse and rat

**Figure 1:**
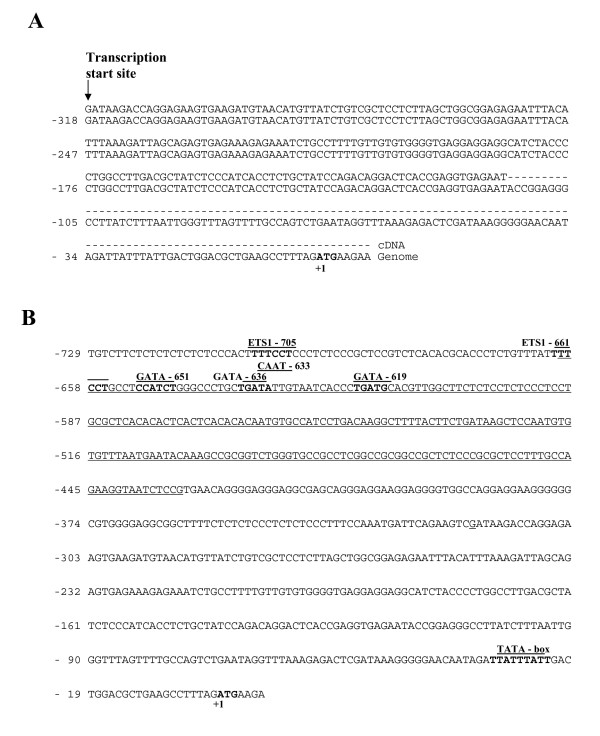
**The sequence of the 5'flanking region of *ETO***. (A) Alignment with genomic DNA of *ETO *cDNA from HEL cells amplified by RLM-RACE. Nucleotide +1 indicates the translational start site (ATG). The transcription start site is shown to be at -318 bp. (B) The sequence of the 5'flanking region of the *ETO *promoter region was amplified by PCR with genomic DNA as template. Putative consensus binding sites for transcription factors identified with MatInspector http://www.genomatix.de/matinspector.html are marked. Alignment of the human sequence with mouse and rat genomic sequences showed a region at -659 to -432 bp (underlined) to be highly conserved suggesting the presence of a proximal promoter.

In order to identify the location of the proximal *ETO *promoter, the transcription start site was identified by use of 5'-rapid amplification of cDNA-ends (RACE) with mRNA extracted from HEL cells. The amplified cDNA was cloned, sequenced and aligned to genomic DNA (Fig. 1A). A complete sequence match with an upstream region of the *ETO *transcript variant-3 (NCBI Ref. Seq: NM_175635.1) was observed and the transcription start site was identified at -318 bp (translational start codon at +1). Orthologous genes may be subject to similar regulatory mechanisms in conserved regions of different species [26]. A search for homologies within the gene upstream of the transcription start revealed a region at -659 to -432 bp that was highly conserved between human, mouse and rat (Fig. 1B). This region may carry important cis-acting regulatory elements. Examination by bioinformatics analysis revealed potential ETS1 binding sites (5'-TTTCCT-3') at -705 and -661; GATA consensus transcription factor-binding sites at -651 (5'-CCATCT-3'), -636 (5'-TGATA-3'), and -619 (5'-TGATGC-3') and a CAAT binding site at -633 (5'-TATTG-3') (Fig. 1B).

### Functional promoter upstream of the ETO coding region with erythroid/megakaryocytic specificity

**Figure 2:**
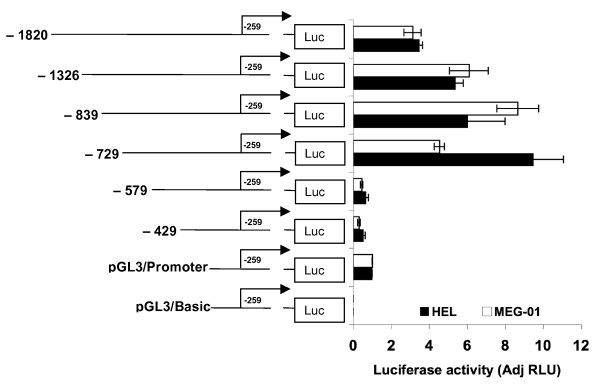
**Effects of 5'deletions on *ETO *promoter activity in erythroleukemia HEL and megakaryocytic MEG-01 cells**. The following reporter constructs were examined: pGL3 -1820-259 (-1820), pGL3 -1326-259 (-1326), pGL3 -839-259 (-839), pGL3 -729-259 (-729), pGL3 -579-259 (-579), and pGL3 -429-259 (-429) after transfection into erythroid (HEL) and megakaryocytic (MEG-01) cell lines. Nucleotide +1 indicates the translational start site (ATG) and nucleotides 5'and 3' thereof have a "-" and "+" designation. The promoterless pGL3/basic and the pGL3/SV40-promoter are used as negative and positive control, respectively. Firefly and Renilla luciferase (internal standard) activities were assayed 24 h post-transfection. The luciferase activity is normalized against pGL3/SV40-promoter activity. The transcriptional activity of the full-length promoter was retained by the -729 to -259 bp region, which is therefore likely to contain the proximal *ETO *promoter. The -579 to -259/-429 to -171 bp regions showed lack of transcriptional activity. Firefly was normalized to Renilla luciferase as internal control for transfection efficiency and the results are given as adjusted Relative Luciferase Units (AdjRLU). Bars represent the mean of results from 3 to 5 separate transfections and the error bars show SEM.

The factors regulating the *ETO *gene expression have to our knowledge not been determined. Therefore, we aimed at identifying major regulatory *cis*- acting regions and *trans*- acting factors regulating human *ETO *expression in hematopoietic cells. To examine whether the sequence upstream of the transcription start of the *ETO *gene is transcriptionally active, we cloned an -1820 to -259 bp region (translational start codon at +1), which was inserted upstream of the luciferase reporter gene in promoterless pGL3/Basic vector, creating the plasmid pGL3/-1820-259. We transfected plasmids into hematopoietic cell lines and determined luciferase activity. Transcriptional activity was normalized to pGL3/SV40-promoter; promoterless pGL3/Basic served as negative control. Renilla vector was used as internal control for transfection efficiency. pGL3/-1820 to -259 showed an approximately 3-fold increased reporter signal compared to pGL3/SV40-promoter both in erythroid (HEL) and megakaryocytic (MEG-01) cell lines (Fig. 2, top) suggesting the presence of strong cis-regulatory elements in this particular region.

In order to identify the functionally important regulatory DNA sequences, sequential deletions were made from the 5'end of the -1820 to -259 bp region. The deletions were inserted upstream of the luciferase reporter gene in promoterless pGL3/Basic thus generating pGL3 -1326-259, pGL3 -839-259, pGL3 -729-259, pGL3 -579-259, and pGL3 -429-259 reporter constructs, which were transfected into erythroid HEL and megakaryocytic MEG-01 cell lines. The region between -729 and -259 bp was found to retain the transcriptional activity (Fig. 2). The -579 to -259 and the -429 to -259 bp regions obtained by further deletions showed no transcriptional activity and may not play a significant role in *ETO *gene expression in HEL or in MEG-01 cells. Hence, the -729 to -259 bp region represents the smallest fragment generated herein that retained full transcriptional activity. Thus, the results from both phylogenetic footprinting and deletional analyses reveal a region, which is likely to contain the proximal *ETO *promoter.

**Figure 3:**
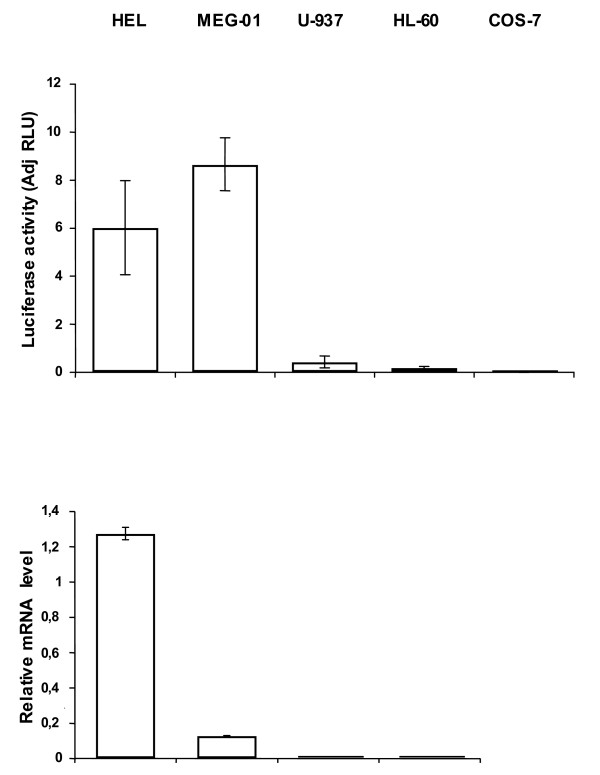
**Relative activity of the proximal *ETO *promoter (-729 to -259 bp) reporter expressed in various cell lines**. The pGL3/basic and pGL3/SV40-promoter are used as negative and positive control, respectively. The luciferase activity is normalized against pGL3/promoter activity. The *ETO *-729 to -259 bp region reporter shows strong activity in the erythroleukemia HEL and in the megakaryocytic MEG-01 cell lines. The myelomonocytic U-937, the promyelocytic HL-60 and the monkey kidney COS-7 cell lines show only low luciferase activity upon expression of *ETO *-729 to -259 bp region. *ETO *transcripts detected by real-time PCR were found in cell lines showing increased luciferase expression upon transfection of the *ETO *-729 to -259 bp region. Lack of ETO expression in COS-7 cells was shown before [54]. Firefly was normalized to Renilla luciferase as internal control for transfection efficiency and the results are given as adjusted Relative Luciferase Units (AdjRLU). Luciferase results are shown for 3 to 5 separate transfections; bars represent the mean and the error bars show SEM. Real-time PCR results are from two experiments in triplicate samples. Relative luciferase unit (RLU) represents experimental value for luciferase activity.

The likely proximal *ETO *promoter region (-729 to -259 bp) was investigated for cell specificity. The pGL3 -729-259 reporter plasmid gave a strong signal in erythroid (HEL) and megakaryocytic (MEG-01) cell lines and a low signal in promyelocytic HL-60, myelomonocytic U-937 and monkey kidney COS-7 cells (Fig. 3). As shown by results from real-time PCR, *ETO *transcripts were detected in the erythroid/megakaryocytic cell lines but not in the myeloid cell lines U-937 and HL-60 or in COS-7 cells (Fig. 3). Thus, even if a limited number of cell lines were investigated a robust correlation is suggested between transfected promoter activity and endogeneous *ETO *gene activity. This result suggests cell-type-specific activation of the *ETO *promoter. However, transfected promoter activity did not correlate directly with the *ETO *mRNA levels detected with real-time PCR. For example, MEG-01 cells showed higher luciferase activities than HEL cells but lower transcript levels (Fig. 3). The relationship between promoter activity and mRNA activity is affected by the endogeneous environment. Gene regulation involves more than promoter activity. Differences in repressor elements could explain the lack of correlation between promoter activity and mRNA.

### Mutagenesis of the GATA -636 consensus binding site represses and mutagenesis of the ETS1 -705 binding site increases transactivation of the ETO promoter in HEL/MEG-01 cells

**Figure 4:**
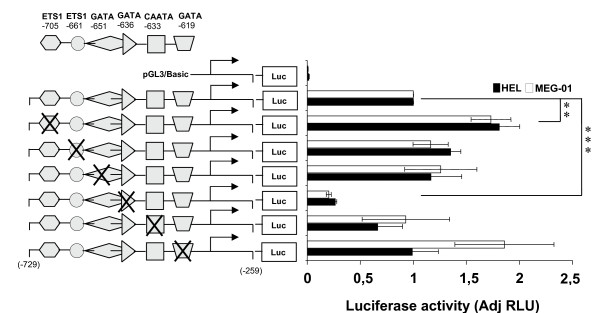
**Mutagenesis of consensus transcription factor binding sequences in the *5'ETO***. Erythroid HEL and megakaryocytic MEG-01 cells were transfected with the -729 to -259 bp *ETO *promoter reporter construct with or without mutations of transcription factor binding sites as indicated (**X**). The mutations are described in "Materials and Methods". The pGL3/basic and pGL3/SV40-promoter are used as negative and positive control, respectively. Firefly and Renilla luciferase (internal standard) activities were assayed 24 h post-transfection. The luciferase activity of mutated ETO promoter is normalized against luciferase activity of wildtype *ETO *promoter. Mutation of the GATA -636 site strongly represses transactivation of the *ETO *promoter reporter. Mutation of the ETS1 -705 binding site increased the luciferase signal twice. Firefly was normalized to Renilla luciferase as internal control for transfection efficiency and the results are given as adjusted Relative Luciferase Units (AdjRLU). Bars represent the mean of results from 3 to 5 separate transfections and the error bars show SEM. **, p < 0.01; ***, p < 0.0001

As mentioned above several potential transcription factor binding sites were detected on the conserved region of the promoter (Fig 1). ETS and GATA factors play a role in erythroid differentiation and CAAT-binding sites are often involved in promoter regulation. Therefore, we choose to determine whether the identified potential transcription factor binding sites of the conserved region contribute to transactivation of the promoter each element was disrupted by site-directed mutagenesis (Fig. 4). Disruption of the GATA -636 binding site led to a 4-fold reduction in reporter gene activity in HEL/MEG-01 cells relative to intact ETO promoter. Conversely, mutation of the ETS1 -705 binding site (outside the evolutionary conserved region) increased the luciferase signal twice. Disruption of the GATA -651, GATA -619, ETS1 -661, and the CAAT -633 binding sites did not significantly affect the reporter signal. CAAT -633 was mutated because CAAT plays a role in promoter regulation. The results suggest that the GATA -636 site may have a role in activation and that the ETS1 -705 sites may have a role in repression of the *ETO *gene activity in cells with erythroid/megakaryocytic potential.

### GATA-1 binds to consensus sites in the ETO promoter *in vitro *and *in vivo *of HEL/MEG-01 cells but not in *in vitro* in G1E cells

**Figure 5:**
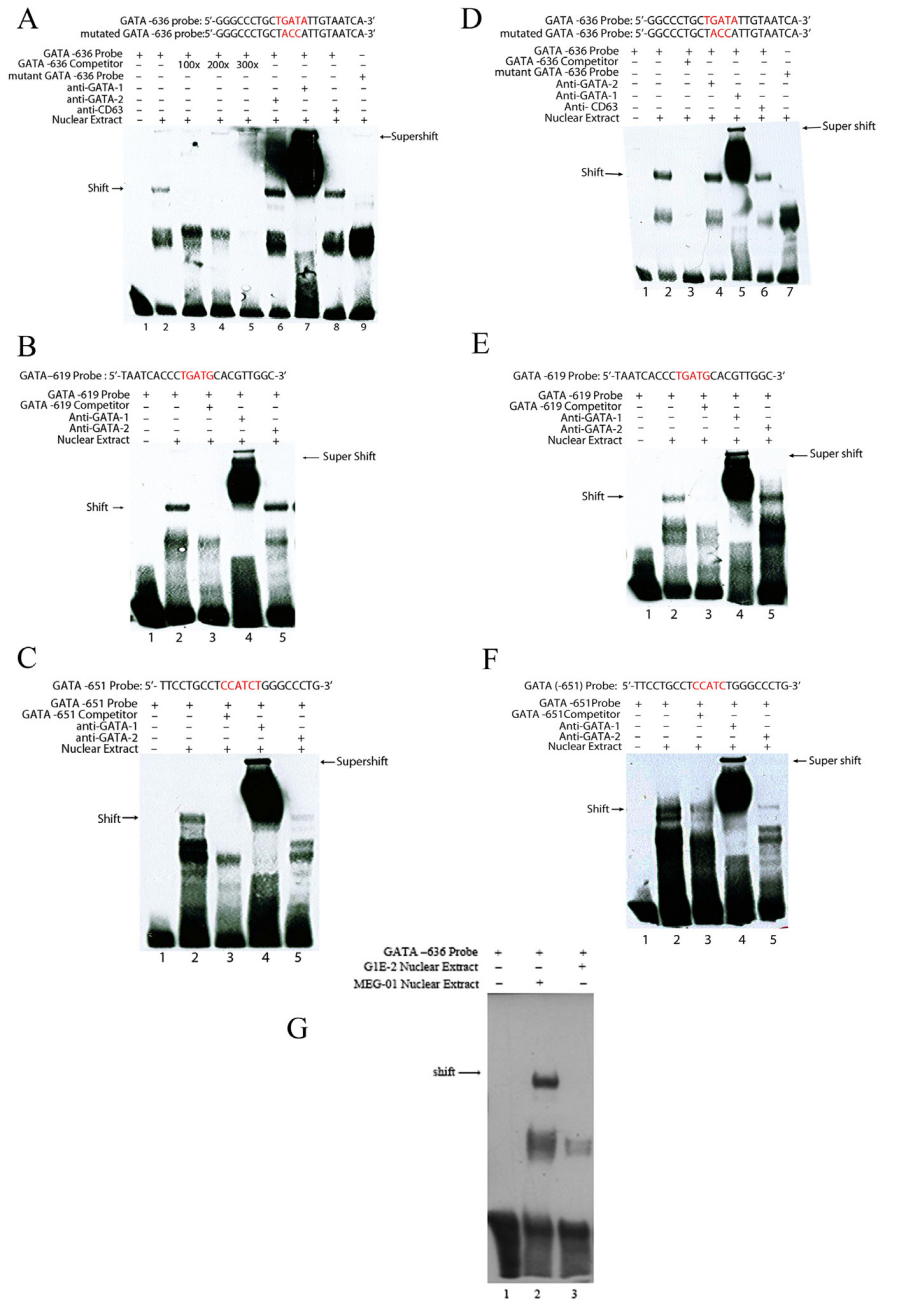
**Detection of DNA-protein interactions using electrophoretic mobility shift/supershift assays in vitro of consensus GATA binding sequences in the 5'promoter of *ETO *and nuclear extracts from HEL/MEG-01/G1E cells**. Sequences for oligonucleotide probes of core consensus and mutated GATA -651, GATA -636 and GATA-619 sites are shown. *Arrows *marked *shift *demonstrate primary DNA-nuclear protein interactions; *arrows *marked *supershift *demonstrate DNA-nuclear protein-antibody interactions. Results for HEL cells (A-C) are to the left, results for MEG-01 cells (D-F) to the right and results for the G1E (G) is at bottom. For the GATA -636 probe a shift is shown in HEL cells (A2) that is competed for by excess unlabelled probe (competitor) (A3-A5) indicating binding of nuclear extract protein to the biotinylated probe that contains the GATA -636 sequence. In support of this no binding was observed to a probe that contains a mutated GATA -636 sequence (A9). Proteins bound to the GATA -636 probe were "super-shifted" by antibody to GATA-1 (A7) but not with antibody to GATA-2 (A6) indicating specificity of the DNA-protein interaction. Similar results are shown for the GATA -636 probe in MEG-01 cells (D). For GATA -619 and -651 probes a shift is shown (B, C, E, F; lane 2) that is competed for by excess unlabeled probe (competitor) indicating binding of nuclear extract protein to the biotinylated probe that contains GATA -619 or -651 sequences. A supershift is shown with antibody to GATA-1 (B, C, E, F; lane 4) but not with anti-GATA-2 (B, C, E, F; lane 5). To try to distinguish between the binding of GATA-1 and GATA-2 to the ETO promoter EMSA was performed with nuclear extract of G1E cells, which lack GATA-1. MEG-01 nuclear extract was used as positive control (G2). No binding of GATA-2 protein to the consensus *ETO *promoter was observed (G3) suggesting lack of GATA-2 interaction. These experiments were repeated twice.

Electrophoretic Mobility Shift Assays (EMSA) and antibody supershift assays were used to examine interactions of the putative GATA binding sequences (GATA -651, GATA -636 and GATA -619) using nuclear extracts from HEL or MEG-01 cells. Biotin-labeled probes, which include the various GATA binding sequences were used for EMSA. Binding of proteins from nuclear extracts to biotinylated probe that includes the GATA -636 sequence was indicated by gel shift (Fig. 5). Specificity of the shift was shown by lack of binding to a probe with mutations within the core consensus sequence and by inhibition of binding of biotinylated probe by excess unlabeled probe. Proteins bound to the GATA -636 probe were "super-shifted" by antibody to GATA-1 but not by antibody to GATA-2 indicating binding of GATA-1 to the consensus site (Fig. 5). GATA -619 and -651 probes also showed a shift that was competed for by excess unlabeled probe indicating specific binding of nuclear extract protein (Fig. 5). Furthermore, proteins bound to the GATA -651 and GATA -619 elements were "super-shifted" by antibody to GATA-1 but not by antibody to GATA-2 (Fig. 5). This indicates that GATA-1 can bind to all three GATA consensus sites within the conserved region of the ETO promoter.

The lack of GATA-2 binding to the -636 probe could result from competition from GATA-1. To determine whether GATA-2 binding can be competitively inhibited by GATA-1, EMSA was performed with nuclear extract of the G1E cell line, which is GATA-1^- ^(null) but GATA-2^+ ^[27]. No primary interaction of GATA-2 with probe that included the GATA -636 sequence was seen (Fig 5G, Lane 3). In lack of primary GATA-2 protein-DNA interaction no supershift was observed with anti-GATA-2 (not shown). The results argue against strong binding of GATA-2 to the -636 site, and therefore do not support competition from GATA-1 for GATA-2 binding, although this can not be entirely ruled out for the experiments with MEG-01/HEL cells.

An additional band besides the one labeled "shift" is present in the EMSA experiments of Fig. 5. It is still present with the GATA mutant oligo (Fig 5A, lane 9) indicating that it is non-specific, but the band is lost by supershift (Fig 5A, lane 7), suggesting that it contains GATA-1 protein. It may represent a non-specific GATA-1 interaction.

**Figure 6:**
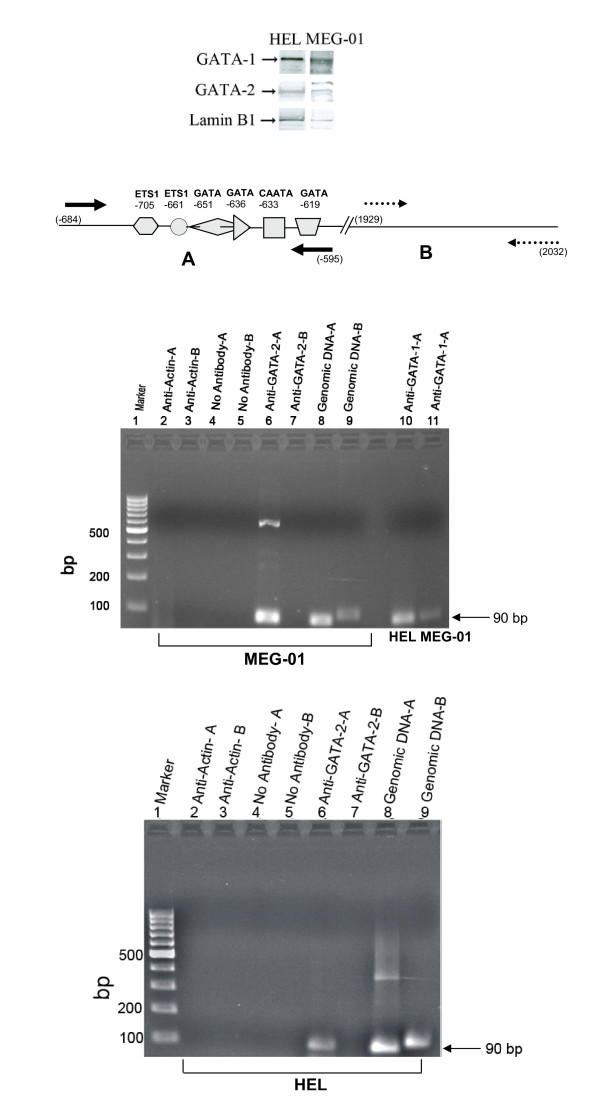
**Chromatin immunoprecipitation (ChIP) assay for examining interactions in vivo of consensus GATA binding sequences in the 5'promoter of *ETO***. The forward and reverse primers used to amplify the proximal promoter region from -684 to -595 (primers **A**, solid arrows) and forward and reverse primers for a downstream region from 1929 to 2032 as control (primers **B**, dashed arrows) are shown. ChIP assays were carried out as described in Methods using chromatin isolated from HEL and MEG-01 cells. PCR products were separated on a 2% gel and representative results are shown. Top and bottom gel figures represent MEG-01 and HEL cells, respectively, except that lane 10 in top gel represents HEL cells. Lane 1, 100-bp ladder; lane 2, actin antibody and primers **A**; lane 3, actin antibody and primers **B**; lane 4, no antibody and primers **A**; lane 5, no antibody and primers **B**; lane 6, GATA-2 antibody and primers **A**; lane 7, GATA-2 antibody and primers **B**; lane 8, genomic DNA and primers **A**; lane 9, genomic DNA and primers **B**. GATA-1 precipitated chromatin amplified with primers **A **in HEL and MEG-01 cells is shown in lane 10 and lane 11, respectively (top gel). By using primers specific for the evolutionary conserved region of the *ETO *promoter, a PCR product is generated both from the anti-GATA-1 and the anti-GATA-2 immunoprecipitated chromatin. No amplification is seen in the absence of antibody or in the presence of anti-actin. The experiment was repeated twice.

Chromatin immunoprecipitation (ChIP) assays were used to examine *in vivo *binding of GATA-1 and GATA-2 to the putative *ETO *gene promoter. ChIP assays were performed using chromatin isolated from HEL or MEG-01 cells and antibodies towards GATA-1 or GATA-2. The presence of GATA-1 and GATA-2 in HEL and MEG-01 cells was confirmed by Western blotting (Fig. 6). The precipitated DNA was examined by PCR amplification of the ETO promoter fragment using gene specific oligonucleotides. By using primers specific for the evolutionary conserved region of the *ETO *promoter, PCR products of 90 bp were generated from the anti-GATA-2- and anti-GATA-1-immunoprecipitated chromatin of MEG-01 cells (Fig 6, top, lanes 6 & 11). ETO promoter amplification with anti-GATA-2 was also obtained in HEL cells (Fig 6, bottom, lane 6). ChIP with anti-GATA-2 and amplification of control region with primers B was negative in both MEG-01 (top, lane 7) and HEL cells (bottom, lane 7) and amplification with control primers B was also negative with anti-GATA-1 in both HEL and MEG-01 cells (not shown). ChIP controls with anti-actin or without antibody were negative (both panels, lane 2-5), while positive PCR controls with genomic DNA were positive (both panels, lanes 8-9. Thus, specific amplification was achieved by precipitation with anti-GATA-1 or anti-GATA-2 only. No amplification was seen in the absence of antibody or in the presence of anti-actin antibody.

In conclusion, results from EMSA/supershift assays demonstrate GATA-1 binding *in vitro *to the GATA -636 binding site supported by ChIP assays demonstrating binding *in vivo *of GATA-1 to the putative *ETO *promoter. These results are consistent with a function of GATA-1 in activation of the ETO promoter suggested by the results of the mutagenesis studies depicted in Fig. 4.

### Overexpression of GATA-1 stimulates the *ETO *promoter

**Figure 7:**
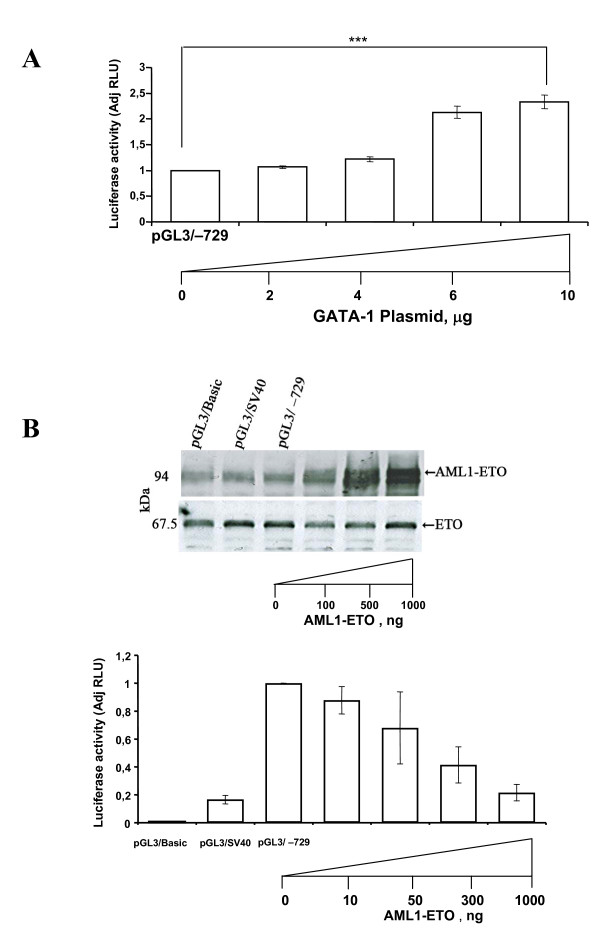
**Effects of overexpression of GATA-1/GATA-2 or AML1-ETO on the *ETO *promoter reporter**. (A) HEL/MEG-01 cells were co-transfected with 15 μg ETO -729 to -259 bp promoter plasmid and 0 to 10 μg of GATA-1 plasmid. The luciferase activity is normalized against the ETO -729 to -259 promoter. The ETO promoter is activated by overexpression of GATA-1 in a dose-dependent manner. Similar results were obtained by co-transfection of HEL cells (data not shown). (B) MEG-01 cells were co-transfected with 15 μg *ETO *-729 to -259 bp promoter plasmid and 0 to 1 μg of AML1-ETO plasmid. The pGL3/basic and pGL3/SV40-promoter are used as negative and positive control, respectively. The luciferase activity is normalized against *ETO *-729 to -259 bp promoter. The *ETO *promoter is strongly repressed in a dose-dependent manner by AML-ETO. Western blotting shows exogeneous AML1-ETO (detected with anti-MTG) and endogeneous ETO expression (detected with anti-ETO). These experiments were repeated three times with similar results. Firefly was normalized to Renilla luciferase as internal control for transfection efficiency and the results are given as adjusted Relative Luciferase Units (AdjRLU). ***, p < 0.0001

GATA-1 was transiently overexpressed in HEL/MEG-01 cells to determine the effect on co-transfected *ETO *-729 to -259 bp promoter. The *ETO *promoter was stimulated in a dose-dependent manner by GATA-1 (Fig. 7A). This result is consistent with a role of GATA-1 in transactivation of the promoter.

### Expression of AML1-ETO represses the *ETO *gene reporter in HEL/MEG-01 cells

AML1-ETO was transiently expressed in HEL/MEG-01 cells to determine the effect on the co-transfected *ETO *-729 to -259 bp proximal promoter reporter. The *ETO *promoter reporter was strongly repressed in a dose-dependent manner by expression of AML-ETO (Fig. 7B)

## Discussion

The goal of this work was to feature the promoter of the *ETO *co-repressor gene. To this end, we identified essential cis-acting elements and *trans*-acting factors that govern *ETO *expression within hematopoietic cells. We identified the likely proximal promoter of the *ETO *gene whose expression within hematopoiesis seemed to be restricted to erythroid/megakaryocytic cells. Examination for regulatory cis-elements of a 1.5-kb region upstream of the transcription start site of the 5' flanking region of the *ETO *gene revealed an initial 400 bp stretch to be required for maximal *ETO *promoter reporter signal when examined in erythroid/megakaryocytic cell lines, which have endogeneous *ETO *expression. Conversely, the *ETO *promoter gave no reporter signal when examined in hematopoietic cell lines with lack of endogeneous ETO. Phylogenetic footprinting revealed a 196 bp region at -659 to -462 bp (+1 indicates translational start codon) containing *cis*-acting elements with GATA binding sites required for regulation of *ETO *transcription.

Disruption of the GATA -636 site within the conserved region repressed transactivation whereas disruption of the ETS1 -705 binding site activated the *ETO *promoter. Examination *in vitro *with EMSA revealed binding of GATA-1 but not of GATA-2 to a probe that included GATA -636 site sequences, the disruption of which abolished transactivation of the *ETO *gene. Our demonstration that EMSA from the G1E cell line, which expresses GATA-2 but lacks GATA-1, also did not show GATA-2 binding to the -636 site, suggests that GATA-2 does not bind to this site. However, the lack of binding of GATA-2 to this probe is of uncertain significance and it is not possible to definitely distinguish between GATA-1 and GATA-2 interactions at the ETO promoter. Examination with ChIP assay revealed binding *in vivo *of GATA-1 to elements within the conserved region of the *ETO *promoter. Furthermore, the promoter was stimulated by overexpression of GATA-1. Collectively, our results demonstrate that the GATA-1 transcription factor binds to the *ETO *proximal promoter and is involved in *ETO *gene expression. The GATA-1 transcription factor is a master regulator in erythroid/megakaryocytic development [28,29].

### Role of GATA-1/ETO in hematopoiesis

GATA-1 belongs to a family of GATA transcription factors, which bind to DNA sequences within the internal GATA-motif A/T(GATA)A/G [30]. GATA-1/GATA-2 recognize similar DNA-binding motifs; their expression profiles overlap for example in the erythroid lineage. GATA-1-mediated *ETO *activation is in agreement with GATA-1 being a critical direct repressor of several target genes including *GATA-2 *[31,32], the repression of which facilitates erythroid differentiation [31,33]. In addition to the cis-acting GATA elements, a putative ETS1 binding element was also identified within the conserved region of the ETO proximal promoter and shown to mediate suppressor activity. Many members of the ETS family for example PU.1, Fli1 and ETS1 are known to play an important role in megakaryocytic and erythroid differentiation [34].

The GATA family of transcription factors contains important regulators of gene expression in hematopoietic cells [35,36]. GATA-1 is essential for the development of early and definitive erythropoiesis/thrombopoiesis [28,29]. GATA-1 deletion results in blocked terminal erythroid and megakaryocytic maturation [37-39]. GATA-1 and GATA-2 are expressed reciprocally during erythropoiesis, GATA-1 levels rise when GATA-2 levels decrease [40]. What role does ETO have in erythroid/megakaryocytic development and differentiation? The regulation of the *ETO *promoter by GATA-1 suggests a role of ETO-mediated gene suppression at a phase of erythropoiesis/thrombopoiesis when GATA-1 is up [41,42]. GATA-1 is expressed at high levels during terminal maturation of erythroid/megakaryocytic cells [43]. Thus, ETO-mediated gene suppressor action may have a role during terminal erythroid/megakaryocytic maturation as a result of GATA-1-mediated *ETO *transactivation.

GATA-1 has a role in erythroid/megakaryocytic cell proliferation and differentiation [44] by activating erythroid-specific genes [33] or megakaryocyte-specific genes [38,39,45] and repressing genes associated with proliferation [31,33,46,47]. The expression level of one member of the ETO homologues, murine MTG16 (ETO2) has already been shown to regulate expansion of erythroid progenitors [3]. Likewise, ETO2 expression in megakaryocytic cells is restricted to immature megakaryocytes and restrains their differentiation [48]. Therefore, ETO2 is suggested to repress inappropriate early expression of terminal megakaryocyte genes by binding to GATA-1 [48]. We have observed that MTG16 decreases during early *in vitro*-induced human erythropoiesis whereas ETO is increased transiently during the peak of erythropoiesis [19]. Therefore, it is possible that ETO, in contrast to MTG16 (ETO2), has a role in repressing genes associated with self renewal and proliferation and that GATA-1-activation of the *ETO *gene might be viewed in this context.

### ETO homologue functions

The ETO homologues are expressed in hematopoietic cells in a more or less cell-type-specific manner [19]. This is supported by the present work, which indicates differences in promoter regulation among the ETO homologues as a possible explanation for lineage-specific expression. We find that the *ETO *promoter is regulated by cis-acting elements contained within an evolutionary conserved region, which is lacking in the 5' flanking region of both *MTG16 *and *MTGR1 *(in silico, data not shown). The 5' flanking region of *MTGR1 *contains an evolutionary conserved region lacking in the two other ETO homologues (in silico, data nor shown). The cell-type-specific hematopoietic expression of *ETO *is much tighter than that of *MTG16 *and *MTGR1 *suggesting specific ETO functions. However, even though their genes are differently regulated, the ETO homologues could have redundant functions if they are expressed in the same cell-type-specific context.

### Suppression of ETO promoter by AML1-ETO

The AML1-ETO fusion protein, which is a gene product of the (8;21) chromosomal translocation of acute leukemia [14,15], binds the promoter region of many genes mostly causing transcriptional suppression [49]. However, some genes regulated by AML1-ETO do not show binding of the fusion protein to the promoter, the transactivation of which is instead affected indirectly [49,50]. The AML1-ETO-mediated suppression of the ETO promoter observed is unlikely to be due to a direct competition for AML1-binding sites, which are not detectable on the promoter. Nevertheless, our observation may be relevant to the reported AML-ETO-induced block of erythroid development [23,24]. If ETO is normally involved in repressing genes associated with self renewal and proliferation, suppression of the *ETO *gene by AML1-ETO could facilitate the AML1-ETO-induced block of erythroid lineage commitment.

## Conclusion

In conclusion, we report that the GATA-1 transcription factor binds to the *ETO *proximal promoter and transactivates the gene in cells of erythroid/megakaryocytic potential in a cell-type-specific manner. The same *trans*-acting factors that are essential in *ETO *expression are essential in erythroid/megakaryocytic differentiation.

## Methods

### Cell culture

The human myelomonocytic U-937, human erythrolukemic HEL, megakaryocytic MEG-01, and promyelocytic HL-60 cell lines were maintained in RPMI-1640 medium supplemented with 10% Fetal Bovine Serum (FBS) (Gibco BRL, Life Technologies, Rockville, MD, USA). Monkey kidney COS-7 cells were maintained in DMEM medium with 10% FBS supplemented with high glucose (4.5 g/l) and L-glutamine. The G1E erythroid cell line, derived from murine embryonic stem cells [27], was maintained in IMDM, Pen/Step 20 ml/L, monothioglycerol 12 μl/L, FCS 20%, human erythropoietin 2U/ml, Stem Cell Factor 50 ng/ml. GIE cells express GATA-2 mRNA at a high level relative to the expression in wild-type proerythroblasts or erythroleukemia cells but are GATA-1^-^(null) [27].

### 5'-rapid amplification of cDNA-ends (RACE)

The mRNA was prepared from HEL cells using Oligotex Direct mRNA mini Kit (Qiagen, Hilden, Germany). The 5'-end (transcription start) of the mRNA was identified by the first choice RNA Ligase mediated (RLM)-RACE kit (Ambion Inc., TX, USA). Nested PCR of the RACE reaction was performed with adapter primer 5'-CGCGGATCCGAACACTGCGTTTGCTGGCTTTGATG-3' and nested gene specific primer 5'-ACTGGTTCTTGGAGCTCCTTGAGTAGT-3'. RACE- products were cloned into pGEM-T Easy Vector system (Promega Corporation, WI, USA) and sequenced.

### Amplification of ETO promoter region

A 5' flanking region of 2049 bp from -2307 to -259 bp was amplified from human genomic DNA by PCR. The forward and reverse primers used were 5'-TGGAGAAATATTACCTTGTTCTCGTCTCAG-3' and 5'-ACACAACAAAAGGCAGATTTCTCTTTCTCAC-3', respectively. Regions corresponding to -1820 to -259, -1326 to -259 and -839 to -259 bp were amplified from the 2049 bp fragment by nested PCR with forward primers 5'- TAGCTC**GGTACC**ACTTTCTGGCCCCATCC-3' (KpnI restriction site highlighted in bold),5'- AGCA**GGTACC**TAAGAGTCACCTGTGGCTAC-3' (KpnI restriction site highlighted in bold), 5'-ACAGCA**GCTAGC**GCTACAGTGCACACCATG-3' (Nhe I restriction site highlighted in bold) and a common reverse primer 5'-TCCGCC**AGATCT**GAGGAGCGACAGATAAC-3' (BglII restriction site highlighted in bold). Sequential 5' deletions of the -1820 to -259 bp promoter region were generated by PCR from the cloned genomic DNA as template to generate -729 to -259, the -579 to -259 and the -429 to -259 bp regions. Forward primers were 5'-AACA**GGTACC**GGAGGCGAGCAGGGAGG-3', 5'-ACAC**GGTACC**ACACACAATGTGCCATCCTG-3' and 5'-TGTCT**GGTACC**TCTCTCTCCCACTTTTCC-3' with KpnI restricton sites highlighted in bold. The common reverse primer is the same as used for amplification of the -2049 to -259 bp region. All sequences were verified.

### Site-directed mutagenesis of transcription factor-binding sites

Oligonucleotide primers including desired mutations were synthesized and used in two-step spliced overhang extension PCR. The following potential transcription factor binding sites were mutated: ETS1 sites at positions -705 and -661, 5'- TCC-3' changed to 5'-GAA-3'; GATA site at position -651, 5'-ATC-3'changed to 5'-GCT-3'; GATA site at position -636 , 5'-GAT-3' changed to 5'- ACC-3'; CAAT site at position -633, 5'-ATT-3' changed to 5'-GCC-3'; GATA site at position -619, 5'-GAT-3' changed to 5'-AGC-3'. After subcloning into promoterless pGL3/Basic reporter plasmid, the mutations were verified by sequencing.

### Luciferase reporter assays

PCR products were cloned into promoterless pGL3/Basic reporter plasmid employing firefly luciferase as specific reporter to generate pGL3 -1820-259, pGL3 -1326-259, pGL3 -839-259, pGL3 -729-259, pGL3 -579-259, and pGL3 -429-259 reporter constructs. The mutants were cloned into the same reporter plasmid. Transient transfections of hematopoietic cell lines were performed by electroporation as previously described by Lennartsson et al [51]. The pGL3/SV40-promoter vector served as positive control and promoterless pGL3/Basic vector as negative control. Renilla luciferase was used as internal control for transfection efficiency. Thirtyfive μg pGL3 DNA were used for HL-60 target cells and 15 μg for U-937, HEL or MEG-01 target cells. At 24 h after transfection cells were disintegrated in 200 μl lysis buffer. Twenty μl triplicate lysate samples were used for luciferase assays with the Dual luciferase reporter assay kit (Promega Corporation, WI, USA). Onehundred μl each of firefly and Renilla substrates were added. Light emission was quantified using standard procedures (Run Promega Protocol, DLR-0-INJ) on the GLOMAX 20/20 Luminometer. Firefly was normalized to Renilla luciferase as internal control for transfection efficiency and the results are given as adjusted Relative Luciferase Units (AdjRLU). Three to five independent transfections were performed in each case.

### Electrophoretic Mobility Shift Assay (EMSA)

Three potential GATA sites for positions -651, -636 and -619 were examined by electrophoretic mobility shift assay (EMSA). The probe sequences were biotin-5-TTCCTGCCT***CCATC***TGGGCCCTG-3', biotin-5'-GGGCCCTGC***TGATA***TTGTAATCA-3' and biotin-5'-TAATCACCC***TGATG***CACGTTGGC-3', respectively. Nuclear extracts from HEL and MEG-01 cells were prepared as described by Andrews and Faller [52]. Three to four μg of nuclear extract were incubated with biotin-labeled probe for 20 min at room temperature with LightShift EMSA optimization kit reagents (Pierce, IL, USA, cat.no. 20148X) as per manufacturer's instruction. Two to four μl polyclonal anti-GATA-1 (Active Motif, Carlsbad, CA, USA), monoclonal anti-GATA-2 (Santa Cruz Biotechnology Inc., CA, USA, sc-9008) or polyclonal anti-CD63 (Santa Cruz Biotechnology Inc., CA, USA, sc-7080) antibodies were added to the reaction mixtures and incubated for 15 min at room temperature. A 20 μl binding reaction mixture contained 1x binding buffer, 2.5% glycerol, 5 mM MgCl_2_, 50 ng/ul poly(dI.dC), 0.05% NP-40, and 20 fmol biotin leveled probe. The samples were separated on a 6% DNA retardation gel (Invitrogen, UK) in 0.5% TBE buffer at 90V followed by semi-dry blotting to 0.45 mm Biodyne B pre-cut modified nylon membranes (Pierce, IL, USA) for 30 min at 20V. Immediately after blotting, DNA was cross-linked to the membrane in the GS gene linker UV chamber (Bio-Rad, CA, USA) for 55 sec (120 mJ/cm^2^). The membrane was processed as per manufacturer's instruction and the chemiluminescence was determined on Hyperfilm ECL (Amersham Pharmacia, UK).

### Chromatin Immunoprecipitation (ChIP) assay

ChIP was performed by use of an IP assay kit (Millipore, MA, USA). Chromatin was prepared from 10^6 ^HEL/MEG-01 cells and cross-linked with 1% formaldehyde at 37°C for 10 min. Cells were washed in ice-cold PBS lacking Ca^2+ ^& Mg^2+ ^and supplemented with protease inhibitor (Roche Applied Science, IN, USA). The cell pellet was resuspended in 200 μl SDS lysis buffer supplemented with protease inhibitor and incubated on ice for 10 min. Sonication was performed on ice for 3-4 sets of 10 sec pulses at 40% amplitude using UP 50 Ultraschallprozessor (LabVision, GmbH) at an interval of 2 min. To reduce non-specific background, sonicated samples were pre-cleared with salmon sperm DNA/protein A agarose slurry. For IP, 4 μl of polyclonal anti-GATA-1 (Active Motif, Carlsbad, CA, USA) or 0.8 mg monoclonal anti-GATA-2 antibodies (Santa Cruz Biotechnology Inc., CA, USA, sc-9008) were added followed by rotation overnight at 4°C. Then, 60 μl salmon sperm DNA/protein A agarose slurry was added and incubated for one h at 4° C with rotation. Agarose-immunoprecipitate was collected by centrifugation and washed as per manufacturer's instruction. Histone complex was eluted with 250 μl freshly prepared elution buffer (1% SDS, 0.1M NaHCO3). Histone DNA crosslinks were reversed at 65° C for 4 h in 5M NaCl followed by digestion with proteinase K. DNA was extracted with phenol-chloroform-isoamylalcohol. The recovered DNA was used in duplicate PCR reactions performed on each immunoprecipitated template. Forward and reverse primers for GATA sites were 5'-TCTCACACGCACCCTCTGTTTATTTTCCTGC-3' and 5'- AGAGGAGAGAAGCCAACGTGCATCAGGGTG-3'. Control forward and reverse primers were: 5'- TCTGCTCCAATATGAATATTGAACTACTTC-3' and 5'- TTGTTTTTAAATAACCCACTCACATTAACA-3'. Three different chromatin preparations were used for each IP.

### Quantitative real-time PCR

Real-time PCR was performed as described previously [19]. Based on the Ct values of the samples, transcript levels were calculated from a standard curve. Relative quantification based on the ΔCt method [53] was used. Normalization: ΔCt = Ct (sample) - Ct (HEL cells of corresponding dilution concentraction). Relative quantification = 2^**-ΔCt**^. Relative mRNA level presented is relatively quantified, subtracted Ct value of HEL cells from the samples Ct value of the corresponding dilution concentration.

### Immunoprecipitation (IP) and Western blotting

IP and Western blotting were performed as described previously [18]. The following antibodies were used: polyclonal anti-GATA-1 (Active Motif, Carlsbad, CA, USA), polyclonal anti-GATA-2 (R&D Systems, MN, USA), polyclonal anti-ETO specifically reactive with ETO [54], and polyclonal anti-MTG reactive with all ETO homologues and AML1-ETO [54].

### Bioinformatics

Sequences of cDNA were analyzed using the NCBI Blast program http://www.ncbi.nlm.nih.gov/BLAST/. Conserved regions were searched by multiple alignment of genomic sequences using ClustalW http://www.ebi.ac.uk/Tools/clustalw2/index.html. Potential transcription factor binding sites were identified with MatInspector http://www.genomatix.de//matinspector.html and the Jaspar database (Jaspar.genereg.net).

### Statistical analysis

The statistical significance between two samples was determined by student's t-test.

## Authors' contributions

RA carried out most of the experiments, analyzed data and was involved in drafting the manuscript. RSD initiated the project, carried out some experiments, supervised experimental design/data analysis and was involved in drafting the manuscript. UG supervised experimental design/data analysis and was involved in drafting the manuscript. IO supervised experimental design/data analysis and was involved in drafting the manuscript. All critically revised and approved the final manuscript.
